# Chimeric antigen receptor T-cells: a review on current status and future directions for relapsed/refractory multiple myeloma

**DOI:** 10.3389/fonc.2024.1455464

**Published:** 2024-08-08

**Authors:** Issam S. Hamadeh, Reed Friend, Sham Mailankody, Shebli Atrash

**Affiliations:** ^1^ Clinical Pharmacy Services, Pharmacy Department, Memorial Sloan Kettering Cancer Center, New York, NY, United States; ^2^ Plasma Cell Disorders Division, Department of Hematologic Oncology & Blood Disorders Levine Cancer Institute, Atrium Health, Charlotte, NC, United States; ^3^ Myeloma Service, Division of Hematologic Oncology, Department of Medicine, Memorial Sloan Kettering Cancer Center, New York, NY, United States

**Keywords:** BCMA, CAR T-cells, T-cell redirection therapies, myeloma, immune therapy

## Abstract

Although multiple myeloma is an incurable disease, the past decade has witnessed significant improvement in patient outcomes. This was brought about by the development of T-cell redirection therapies such as chimeric antigen receptor (CAR) T-cells, which can leverage the natural ability of the immune system to fight myeloma cells. The approval of the B-cell maturation antigen (BCMA)-directed CAR T, idecabtagene vicleucel (ide-cel), and ciltacabtagene autoleucel (cilta-cel) has resulted in a paradigm shift in the treatment of relapsed/refractory multiple myeloma. Overall response rates ranging from 73 to 97% are currently achievable. However, the limitations of KarMMa-1 and CARTITUDE-1 studies spurred the generation of real-world data to provide some insights into the effectiveness of ide-cel and cilta-cel among patients who were excluded from clinical trials, particularly those who received prior BCMA-targeted or other T-cell redirection therapies. Despite their unprecedented clinical efficacy in heavily pretreated patients, responses to CAR T remain non-durable. Although the underlying mechanisms of resistance to these agents haven’t been fully elucidated, studies have suggested that resistance patterns could be multifaceted, implicating T-cell exhaustion and tumor intrinsic mechanisms such as BCMA target loss, upregulation of gamma-secretase, and others. Herein, we provide a succinct overview of the development of CAR T-cells, manufacturing process, and associated toxicities/complications. In this review, we also recapitulate the existing literature pertaining MM CAR-T as well as emerging data from some of the ongoing clinical trials designed to mitigate the shortcomings of these agents, and improve the clinical efficacy of CAR T, especially in the relapsed/refractory setting.

## 1 Introduction

Multiple Myeloma (MM), a blood cancer that originates from plasma cells in the bone marrow, comprises about 1.8% of all new cancer cases. In 2024, an estimated 35,780 new cases and 12,540 deaths were reported (1, 2). Despite the recent advancements in understanding tumor biology and developing novel therapies, MM remains incurable, characterized by cycles of remission and relapse. With each relapse or progression, the remission period becomes shorter until the disease becomes refractory to the currently available or standard therapies (3).

A multicenter retrospective analysis, which included 275 MM patients, revealed dismal outcomes, particularly in penta-refractory patients (i.e., refractory to two proteasome inhibitors, 2 immunomodulatory agents, and 1 anti-CD38 monoclonal antibody, n=70), where the median overall survival (OS) was about 6 months (4). Such real-world data suggest that there is an unmet need to develop novel agents for heavily pretreated MM patients, which have the potential to alter the natural history of the disease.

Defects or alterations in immune surveillance that occur during tumorigenesis have recently become a topic of extensive research. This culminated in the development of novel therapeutic approaches such as immunotherapies, which can harness the intrinsic power of the immune system to treat the disease. B-cell maturation antigen (BCMA) is a transmembrane glycoprotein belonging to the tumor necrosis factor receptor superfamily 17 (TNFRSF17). Its high expression on myeloma cells made it a valuable and attractive target for MM immunotherapy, particularly in the relapsed/refractory (RR) setting (5). Chimeric antigen receptor (CAR) T-cells targeting BCMA are one type of immunotherapies that can induce both tumor-directed cytotoxicity and immunological memory; they have demonstrated unequivocal efficacy in RRMM.

This review provides a comprehensive overview of the development of CAR T-cells, published data about the clinical efficacy of the approved products, their limitations, and underlying mechanisms of resistance, and some of the investigational platforms currently in development designed to circumvent the shortcomings of the available products.

## 2 Overview of CAR T-cells: CAR structure, classification and manufacturing, administration, and complications

Effective eradication of cancer cells via the immune system is a multi-step process. When cancer cells shed their antigens (including tumor-associated antigens) into the bloodstream, these antigens are taken up by the antigen-presenting cells, such as dendritic cells, which process and present them to the T-cells in the context of major histocompatibility complex (MHC) molecules (6). Of note, both CD8+ and CD+4 T-cells recognize these antigens only when bound to MHC I and II molecules. Upon activation, the T-cells secrete perforins and granzymes that trigger a cascade of reactions leading to apoptosis of the tumor cells. Tumor cells can offset this T-cell-mediated immune response via several mechanisms; one of them is to intrinsically downregulate the expression of MHC molecules (7). Such findings provided the impetus to develop new modalities or approaches that have the potential to mitigate the need for MHC molecules to trigger a T-cell-mediated immune response.

### 2.1 The general structure of CARs

CARs are hybrid receptors that can be genetically engineered/designed and transferred to T-cells (CAR T-cells), thereby allowing the latter to identify specific tumor-associated antigens in a manner that is independent of MHC I and II molecules. From a structural standpoint, the CAR can generally be divided into four main domains or components, each of which plays an essential role in recognizing the target antigen (8).

- An extracellular target antigen binding domain.
- Hinge region (which, together with the extracellular domain, constitutes the ectodomain.
- A transmembrane domain.
- The intracellular signaling domain is also referred to as the endodomain.

As the name suggests, the extracellular target antigen binding domain of the CAR confers target (antigen) specificity, given the fact that it is usually derived from the variable heavy (V_H_) and light (VL) chains of monoclonal antibodies of mouse origin. A flexible linker connects the V_H_ and V_L_ chains to each other to form the so-called single chain variable fragment (scFv) (9). This allows the CAR to recognize extracellular antigens, which, in the case of multiple myeloma, is mainly the B-cell maturation antigen (BCMA); however, multiple myeloma cells express on their surfaces a multitude of other antigens (GPCR5, CD38, FcRH5, etc.) that can be targeted by specific CARs.

The hinge region, also known as the “spacer, “ serves as a bridge connecting the scFv portion to the transmembrane domain. The hinge region imparts flexibility, allowing the antigen binding domain to readily access the targeted epitope without any steric hindrance, forming a synapse with the antigen. The longer the hinge region, the more flexibility the CAR has (10).

The transmembrane domain helps anchor the CAR to the cell membrane of the T-cells through a hydrophobic α helix. Although it is the least studied component, the transmembrane domain is essential for the stability and function of the CAR-T cell (11).

The internal signaling domain is the most distal intracellular portion of the CAR; it mainly consists of CD3ζ sequences that harbor the immunoreceptor tyrosine-based activation motifs (ITAMS) which become phosphorylated when the CAR binds to its target antigen. Hence, the internal domain is responsible for signal transmission into the cell interior (12).

CARs that consist of the antigen binding domain (scFv) and CD3ζ (intracellular domain) are often referred to as “first-generation” CARs. These have fallen out of favor due to their modest clinical efficacy, exemplified by their limited activation, expansion, and persistence.

### 2.2 Different generations of CAR T-cells

To overcome these shortcomings, modifications have been made to the structure of the endodomain through the incorporation of costimulatory molecules, which led to the inception of several generations of CAR T-cells.

#### 2.2.1 Second-generation CAR T-cells

In addition to CD3ζ, the intracellular domain contains co-stimulatory molecules such as CD28 and 4-1BB to boost the immune signal. The two FDA-approved CAR T-cell products for multiple myeloma, idecabtagene vicleucel (ide-cel) and ciltacabtagene autoleucel (cilta-cel), have 4-1BB as a co-stimulatory molecule. The main advantages of 4-1BB over CD28 are slower expansion and longer persistence. Because of these properties, 4-1BB containing CAR T-cells are less prone to exhaustion (13, 14).

#### 2.2.2 Third-generation CAR T-cells

These have 2 co-stimulatory domains, 4-1BB and CD28, to amplify the intracellular signaling. Subsequently, third generation CAR T-cells have better expansion and differentiation into memory T-cells (15).

#### 2.2.3 Fourth generation CAR T-cells

They are also referred to as “T-cells redirected for universal cytokine killing, TRUCKs” because they are constructed or designed in a manner where they secrete inflammatory cytokines such as interleukin-2 (IL-12) upon activation of antigen binding domain. This unique characteristic further enhances the proliferation and function of the CAR T-cells (16).

#### 2.2.4 Fifth generation CAR T-cells

They are currently being designed for the sole purpose of improving the safety of the product. Fifth generation CAR T-cells contain more additional intracellular domains and drug-dependent ON or OFF-switches to circumvent some of the “off-tumor” activity of the product (17).

### 2.3 Production/manufacturing of CAR T-cells and their administration for treating patients with multiple myeloma

Engineering/manufacturing of CAR T-cells is complex and involves several phases (18). This journey begins with collection of the patient’s peripheral blood mononuclear cells through leukapheresis. Because the patient’s own cells are utilized, the final product is often referred to as “autologous CAR T-cell therapy” to distinguish it from products where the T-cells are collected from a donor. The T-cells are subsequently selected using anti-CD4 and anti-CD8 microbeads and then activated through various *in-vitro* activation methods to facilitate their *in-vitro* expansion.

Insertion of the CAR into the genome of the T-cells occurs through either viral vectors (retroviral or lentiviral vectors) or transposons followed by expansion in a bioreactor in a medium with anti-CD3/CD28 monoclonal antibodies and cytokines. When the required cell dose is reached, the CAR T-cells are isolated and transferred to a bag, where they are resuspended in an infusion-compatible medium. Because the manufacturing process is lengthy, as described above (it takes about 4-5 weeks), patients with high disease burdens may require bridging chemotherapy before administering CAR T-cells.

Prior to CAR T-cell infusion, patients should receive a lymphodepleting conditioning regimen, which creates a suitable environment for the *in vivo* expansion of these cells. Fludarabine (30 mg/m^2^) in combination with cyclophosphamide (300 mg/m^2^) is the most commonly used preparative regimen in multiple myeloma. The regimen is administered for three consecutive days, i.e., on days -5, -4, and -3, prior to the infusion of the CAR T-cells on day 0.

### 2.4 Major complications of CAR T cells therapy

Some of the acute or early complications with CAR T-cells are cytokine release syndrome (CRS) and neurotoxicity/immune effector cell-associated neurotoxicity syndrome (ICANS) (19). Although the pathophysiology of these syndromes is beyond the scope of this paper, it has been demonstrated that tumor cell destruction following CAR T-cell activation results in a drastic surge in the levels of inflammatory cytokines [interferon-γ, tumor necrosis factor-α (TNF-α), granulocyte-macrophage colony-stimulating factor, interleukin (IL)-10, IL-6 and others]. This high concentration of cytokines generates a systemic inflammatory response or cytokine storm that impairs internal organ function. The cardinal manifestations of CRS are fever, hypotension, and hypoxia (20). The median time to onset of CRS varies with each product as illustrated in **Table 1**. The underlying pathophysiology of neurotoxicity/ICANS remains poorly understood; it has been postulated that disruption of the blood-brain barrier, as well as activation of the endothelial cells, results in T-cell infiltration into the brain parenchyma. In addition, the neurologic complications could exhibit in a biphasic pattern i.e. acute and/or delayed (20). Early signs and symptoms of acute neurotoxicity include confusion, disturbances in writing and language, agitation and obtundation; severe acute events are characterized by seizures and cerebral edema. Delayed neurotoxicity is associated with movement, cognitive and personality changes, and its median time to onset is about 27 days (range: 14-108 days) (25).

**Table 1:** Reported adverse effects with ide-cel and cilta-cel.

Study	CRS	CRS, grades 3/4	Median time to onset	Median duration	ICANS	ICANS grade 3/4	Median time to onset	Median duration
KarMMa-1 (21)	Overall: 84%	Overall: 5%	1 day (range: 1-12)	5 days (range: 1-63)	Overall: 18%	Overall: 3%	2 days (range: 1-10)	3 days (range: 1-26)
	D1: 50%	D1: 0%			D1: 0%	D1: 0%		
	D2: 76%	D2: 6%			D2: 17%	D2: 1%		
	D3: 95%	D3: 6%			D3: 20%	D3: 6%		
CARTITUDE-1 (22)	95%	4%	7 days (IQR: 5-8)	4 days (IQR: 3-6)	21%	9%	8 days (IQR: 6-8)	4 days (IQR: 3-7)
									KarMMa-3 (23)	88%	4%	1 day (range:1-14)	4 days (range: 1-51)	15%	3%	3 days (1–317)	2 days (range: 1-37)
CARTITUDE-4 (24)	76%	1%	8 days (range: 1-23)	3 days (range: 1-17)	21%	8%	10 days (6–15)	2 days (range: 1-6)									

Because timely recognition of CRS and neurotoxicity/ICANS is central to reducing morbidity and mortality, several guidelines were crafted to help with grading and managing these serious complications CAR T-cell therapy (26, 27).

Some of the long-term complications of CAR T-cells include B-cell aplasia and hypogammaglobulinemia, both of which predispose patients to infections, thereby warranting antibiotic prophylaxis and intravenous immunoglobulin support.

## 3 Clinical efficacy of the approved and investigational CAR T-cells for relapsed/refractory multiple myeloma

Ide-cel and cilta-cel are the only two FDA-approved CAR T-cells for treating relapsed/refractory multiple myeloma (18–20). Both target the BCMA antigen, which is heavily expressed on the surface of these cells. From a structural standpoint, ide-cel has a single BCMA binding domain, whereas cilta-cel has two BCMA binding domains.

Based on the pivotal KarMMa-1 and CARTITUDE-1 trials, these products were initially reserved for heavily pretreated MM patients who received at least 4 lines of therapy, including an immunomodulatory agent, proteasome inhibitor and an anti-CD38 monoclonal antibody. The key findings from these trials are summarized in **Table 2**. However, the recent data from the KarMMa-3 and CARTITUDE-4 prompted the FDA in April of 2024 to expand the indications for both ide-cel and cilta-cel and approve them for earlier lines of treatment in patients with RRMM (23, 24). According to the new approval, ide-cel is approved for treating patients who received at least two prior lines of therapy, including a proteasome inhibitor, an immunomodulatory agent, and a CD-38 monoclonal antibody. Cilta-cel can also be considered to treat patients who have received at least one line of therapy, including a proteasome inhibitor and an immunomodulatory agent, and are refractory to lenalidomide.

**Table 2:** Summary of the findings from clinical studies evaluating ide-cel and cilta-cel.

	KarMMa-1 (N=128)	CARTITUDE-1 (N=97)	KarMMa-3 (N=254)	CARTITUDE-4
Patient age, years (range)	61 (33–78)	61 (56-68)	63 (30-81)	61.5 (27-78)
Extramedullary disease, %	39	13	24	21.2
High risk cytogenetic features, %	35	24	42	59.4
High tumor burden, %	51	22	28	20.4
R-ISS stage III, %	16	14	12	5.8
Bridging, %	88	75	84	100
Prior lines of therapy	6 (range: 3-16)	6 (range 4-8)	3 (range: 2-4)	1 (32.7%)
				2 (39.9%)
				3 (27.4%)
CAR- T cells	Idecabtagene vicleucel	Ciltacabtagene autoleucel	Idecabtagene vicleucel	Ciltacabtagene autoleucel
Target dose	D1: 150x10^6^	0.75x10^6^/kg (range: 0.5x10^6^-1x10^6^)	150x10^6^-450x10^6^	0.75x10^6^/kg
	D2: 300x10^6^			
	D3: 450x10^6^			
Overall response rate, %	Overall: 73	97 (95% CI: 91.2-99.4)	71 (95% CI: 66-77)	84.60%
	D1: 50			
	D2: 69			
	D3: 81			
Complete response	Overall: 33	67	39 (95% CI: 33-45)	73.1
	D1: 25			
	D2: 29			
	D3: 39			
MRD at 10^-5^ sensitivity, %	26 (95% CI: 19-34)	27	20 (95% CI: 15.2-25)	60.6
PFS	Overall: 8.8 months (95% CI: 5.6-11.6)	34.9 months (95% CI: 25.2- NE) CR	13.3 months (95% CI: 11.8-16.1)	12 months: 75.9% (95% CI: 69.4-81.1%)
	D1: 2.8 months (95% CI: 1.0- NE)			
	D2: 5.8 months (95% CI: 4.2-8.9)			
	D3: 12.1 months (95% CI: 8.8-12.3)			
OS	19.4 months (95% CI: 18.2- NE)	36 months: Overall: 62.9%		12 months: 84.1%
		≥ CR: 59.8%		
		12-month sustained MRD negativity: NE		
		12-month sustained MRD negativity-CR: NE		
Reference:	(21)	(22)	(23)	(24)

### 3.1 Real-world data for approved CAR T-cells

The stringent inclusion criteria that were set forth in the KarMMa-1 and CARTITUDE-1 studies (inadequate organ function, prior exposure to BCMA-targeted therapies, cytopenias, performance status, etc.) precluded a group of patients from participating in these pivotal studies who resembled those in the real world. Hence, there has been great interest in evaluating/replicating the efficacy of ide-cel and cilta-cel in real-world settings. The study by Hansen et al. included 159 patients who received commercial ide-cel at 11 institutions (median dose: 407.0x10^6^ cells, range:154.1-456.4); the number of prior lines of therapy was 7 (4–18), 21% (n=33) had prior exposure to BCMA-targeted therapies and 46% (n=73) met 2 of the exclusion criteria in the KarMMa-1 study (28). The clinical efficacy was in line with what was previously noted or reported, where the overall response (OR) and at least complete response (CR) rates were 84% and 42%, respectively. The median progression free survival (PFS) was 8.5 months (95% CI: 6.5-NR), and OS was 12.5 months (95% CI: 11.3-NR). In the subgroup of patients who were previously exposed to BCMA-targeted therapies, the ORR (73%) and at least CR rate (33%) did not differ significantly from those who had no prior BCMA-targeted therapies (p=0.2). The PFS differed significantly based on the type of prior BCMA-targeted therapy; The PFS was significantly shorter among the patients who were previously treated with belantamab mafodotin (n=25; 5.3 months, 95% CI: 3.0-NR, p=0.0043) or BCMA bispecific T-cell engager antibody (n=4; 2.7 months, 95% CI: 1.9-NR, p=0.00069). Alternatively, the difference in PFS did not reach statistical significance among those with prior CAR-T cells (n=4, p=0.72). CRS rates occurred in 82% of patients (grade 2: 20%) and ICANS in 18% (grade 2: 11%).

Dima et al. assessed the efficacy of ide-cel as a standard of care (SOC) in 69 RRMM patients who did not meet the eligibility criteria of the KarMMa-1 study at 3 US academic institutions (part of the US Myeloma Innovations Research Collaborative, USMIRC) (29). Compared to KarMMa-1, SOC ide-cel demonstrated improved efficacy with and ORR and at least CR rate of 93% and 48%, respectively. The median PFS was comparable between the two studies, 8.5 months (95% CI, 6.2–10.9). Furthermore, there were 18 patients who were previously treated with BCMA-directed therapies (belantamab mafodotin: 16 and BCMA-directed CAR T-cells: 2). Interestingly, patient outcomes were not impacted by prior treatment with BCMA-directed therapies where the ORR in this subgroup was 90%, at least CR was 47% and median PFS was 6.2 months.

The study by Sidana et al. also provided some insights into the real-world efficacy of ide-cel among 603 patients using the CIBMTR database (30). The number of prior lines of therapy was 7 (4–21), and 5% (n=28) had prior exposure to BCMA-directed CAR T cell therapy. The ide-cel dose ranged from 300-460x10^6^ cells. The ORR was noted in 71% (n=421) of the patients; of whom 27% (n=162) achieved a CR. The 6- month PFS and OS rates were 62% (95% CI: 58-66%) and 82% (95% CI: 79-85%), respectively.

Real-world data for cilta-cel demonstrated an ORR of 80% and a CR rate of 40% among 139 patients with RR disease treated at 12 academic centers in the U.S. 36% (n=50) had penta-refractory and the number of prior lines of therapy was 6 (2–18) (31). The reduced efficacy of cilta-cel in this study could be explained by the higher percentage of patients with extramedullary disease (35% compared to 13% in CARTITUDE-1) and high-risk cytogenetic features (41% vs. 24%).

### 3.2 Efficacy of approved CAR T-cells following antibody-drug conjugates and other bispecific T-cell engager antibodies

Recently, other T-cell redirection therapies or bispecific T-cell engager antibodies (teclistamab, elranatamab, and talquetamab) have been approved for RRMM. Despite the availability of several options to treat patients in this setting, the proper sequencing of these agents (CAR T-cells and bispecific T-cell engager antibodies) has become a dilemma. This has also posed the question of whether CAR-T cells retain their efficacy following BCMA-directed therapies or bispecific T-cell engager antibodies.

Given this gap in our knowledge, Cohen et al. sought to investigate the efficacy of cilta-cel in a small cohort of 20 patients with RRMM who were enrolled in the CARTITUDE-2 study and previously treated with noncellular BCMA-directed therapies (either an antibody-drug conjugate or a bispecific antibody) (32). The authors noted an ORR of 60% (95% CI: 36.1-80.9%), with 30% achieving at least a CR. The PFS was 9.1months (95% CI: 1.5-NE), and OS was not reached at the time of data cut-off. Further stratification based on the type of prior BCMA therapy revealed an ORR of 61.5% (95% CI: 31.6-861%) in the antibody drug conjugate (ADC) exposed group and 57.1% (95% CI: 18.4-90.1%) in the bispecific antibody exposed group. The two main factors that predicted response to cilta-cel were the treatment duration of prior anti-BCMA therapy (29.5 days in responders vs. 63.5 days in non-responders) and the elapsed time between the two treatment modalities (235 days in responders vs. 117.5 days in non-responders). The median PFS appeared to be longer in the ADC exposed (9.5 months, 95% CI:1-NE) relative to the bispecific antibody exposed group (5.3 months, 95% CI: 0.6-NE). Despite the small number of patients, 13 patients with prior BCMA-ADC and 7 patients with prior BCMA-bispecific antibody, these findings suggested that cilta-cel might be considered a viable option for select heavily pretreated MM patients who received prior BCMA-directed therapies, namely anti-BCMA ADC. In a case series of 5 heavily pretreated MM patients who received a median of 7 lines of therapy (range: 5-8) including prior BCMA-directed CAR T-cells (3 investigational CAR T-cells and 2 ide-cel), Attar N et al. reported an ORR rate of 80% (n=4) and a CR of 60% (n=3). The PFS rate at 6 months was 75%. Of note, the time elapsed between the two CAR T-cells ranged from 8 to 38 months (33).

The retrospective study by Ferreri et al, evaluated the clinical efficacy of commercial ide-cel in 50 patients who were previously treated with BCMA-targeted therapies (38 received belantamab mafodotin, 7 bispecific antibody, and the rest investigational CAR T-cells) (34). The median number of prior lines of therapy was 9 (range: 4-18), and 86% (n=43) received bridging therapy before ide-cel infusion, where the median dose was 403.3 x10^6^ (range: 154.1-454). The ORR was 74%, with 29% achieving at least a CR. The median PFS was 3.2 months, and OS has not been reached given the short follow-up period with an estimated 6-month OS rate of 72%. Response stratification based on the type of BCMA-targeted therapy showed that the patients who had prior CAR T-cells had the highest response rate (100%), whereas those who received belantamab mafodotin had the lowest response rate (68%); the ORR among patients treated with bispecific antibodies was 86%. Similarly, patients previously treated with CAR T-cells had a longer PFS (NR) compared to those with prior exposure to belantamab mafoditin (PFS: 3.2 months) or bispecific antibodies (PFS: 2.8 months) (34).

Although these studies were small, they provided evidence of CAR T-cells’ efficacy post other T-cell redirection therapies; nevertheless, the duration of response appeared to be modest compared to what was described in KarMMa-1 and CARTITUDE-1. Larger studies may be warranted to corroborate these findings to help not only select patients who are likely to benefit from these agents, but also guide the optimal sequencing of these therapies.

### 3.3 Investigational CAR T-cells for multiple myeloma: a delve beyond BCMA

While further investigation is underway, **Tables 3**, **4** highlight some of the recent advancements which are exploring additional targets to overcome some of the current limitations of BCMA targeted- CAR T-cells such as antigen escape, relapse, and toxicity. Some promising candidates include GPRC5D, SLAMF7, CD38, CD138, and CD19 **Table 5** and **Table 6**.

**Table 3:** Current or ongoing clinical trials with ide-cel.

	Elranatamab in RRMM	KarMM-7	KarMMa-9
Experimental arm	Elranatamab given after (SOC) Ide-cel	Arm A: Ide-cel + CC-220 (+/- low dose dex) Arm B: Ide-cel + BMS-986405	Ide-cel + Len maintenance
Control arm	NA (single arm study)	NA (single arm study)	Len maintenance
Trial Phase	2	1/2	3
Prior lines of therapy to enroll	≥4 including PI, IMID, anti-CD38 mAb	≥3 for both Arms except Arm A, cohort 2 can receive at least 1 but no >3 LOT	4-6 cycles of induction therapy (incl PI, IMID) and single ASCT 80-120 days prior to consent
Primary end point	CR or sCR post consolidation therapy; PFS	DLT, CRR	PFS
NCT	06138275	04855136	06045806

SOC, standard of care; NA, not applicable; PI, proteosome inhibitor; IMID, immunomodulatory; mAb, monoclonal antibody; CR, complete response; sCR, stringent complete response; LOT, lines of therapy; DLT, dose limiting toxicity; CRR, complete response rate; Len, Lenalidomide; LOT, lines of therapy; PFS, progression free survival.

**Table 4:** Current or ongoing clinical trials with cilta-cel.

	CARTITUDE-5	CARTITUDE-6
Experimental arm	NDMM, transplant deferred, treated with VRd induction followed by cilta-cel	NDMM, transplant eligible, following DVRd followed by cilta-cel
Control arm	VRd followed by Rd maintenance	DVRd followed by ASCT
Trial Phase	3	3
Primary end point	PFS	PFS, sustained MRD neg CR
NCT	04923893	05257083

NDMM, newly diagnosed Multiple Myeloma; VRd, Bortezomib, Lenalidomide, dexamethasone; Rd, Lenalidomide, dexamethasone; DVRd, Daratumumab, Bortezomib, Lenalidomide, dexamethasone; PFS, progression free survival; MRD, minimal residual disease; CR, complete response.

**Table 5:** New targets for CART.

New targets for CART		
Target	Extra properties	Trial
SLAMF7/CS1	incorporating on/off suicide gene	NCT03958656 and NCT03710421
	Off the shelf, UCARTCS1	NCT04142619
CD38	–	NCT03464916
	dual-specificity anti-CD38/BCMA CAR T-cell	NCT03767751
CD19	–	NCT02135406
	dual-specificity anti-CD19/BCMA CAR T-cell	NCT03455972
TACI	Targeting BCMA and TACI	NCT03287804
κ light chain	Costimulating domain CD28	NCT00881920
Lewis Y	Costimulating domain CD28	NCT01716364
NY-ESO-1	34% of the HLA-A2 positive expressed NY-ESO-1 and/or LAGE-1	NCT03638206
NKG2D ligands	Costimulating domain DAP10	NCT02203825

**Table 6:** New CAR-Ts designed to offset resistance mechanisms.

New CAR-Ts designed to offset resistance mechanisms							
Resistance mechanism	Mechanism of action	CAR-T properties	No. Of patients	Median follow up	Response	Toxicity	Reference
Antigen escape	Dual-targeted CAR T- cell therapy	BCMA/CD38 bispecific CAR T-cells	23 R/R MM	9 months	ORR 87%, sCR 52% PR 33%	CRS (87%), ICANS (0%), infections (22%)	(36)
			16 RRMM	11.5 months	ORR 88%, CR 81%, PR 6%	CRS (75%), HLH (6%), infections (38%)	(37)
		BCMA/CS1 bispecific CAR T-cells	16 RRMM	290 days	ORR 100%, sCR 31% PR 13%	CRS (38%)	(38)
		BCMA/GPRC5D bispecific CAR T-cells	Na	Na	Na	Na	NCT05431608
		CD38 and SLAMF7	Na	Na	Na	Na	(39)
	Combined infusion	anti BCMA and anti-CD38 CAR T- cells	22 RRMM	24 months	ORR 91%, CR 55%	CRS (100%), ICANS (14%), infections (17%)	(40)
		anti BCMA and anti-CD19 CAR T- cells	62 RRMM	21 months	ORR 92%, CR 60%, PR 21%	CRS (95%), ICANS (11%), B cell aplasia (30%), infections (45%)	(41, 42)
		anti BCMA and anti-CD19 CAR T- cells and maintenance	10 RRMM 20 high risk	Range: 248- 966 days	ORR 23%, CR 6% PR 6%	CRS (90%), ICANS (3%)	(43)
		Anti-BCMA and anti-CD19 CAR T- cells followed by lenalidomide after auto-HSCT	10	42 months	ORR 100%, sCR 90%, CR 10%	CRS (100%), infections (100%)	(44)
		Combined infusion of anti-BCMA and anti-CD19 FasT CAR T-cells	13 high-risk NDMM	5.3 months	ORR 95% sCR 69%	CRS (23%)	(45)
		Combined CAR T-cells with gamma secretase	18 RRMM	10 months. sBCMA levels ≤ 3.0 ng/mL (low) at day 60 PFS of 31 months vs. (high) PFS of 5.4 months	ORR 89%, sCR 44%	CRS (94%) ICANS (39%)	(46)
	Different target	anti-GPRC CAR T-cell (OriCAR-017)	10 RRMM	238 days	ORR 100% sCR 60% 40% VGPR	CRS(100%), no ICANS	(47)
		anti-GPRC CAR T-cells (MCARH109)	17 RRMM	10 months	ORR 70%	CRS (88%), ICANS (6%), Cerebellar disorder (12%)	(35)
CAR T-cell exhaustion	Allogeneic BCMA-targeting CAR T-cells	ALLO-715	43 RRMM	10 months	ORR (56%), with dose: 320 × 106 CAR+ T (n = 24), ORR (71%), VGPR (45.8%), CR (25%)	CRS (55%), infections (44%)	(48, 49)
		P-BCMA-ALLO1	24 RRMM	–	ORR Post-BCMA (60%)	CRS (14%), ICANS (4%)	(50, 51)
	Enrich for memory-like T cells	bb21217	72 RRMM	9 months	ORR (69%)	CRS (75%), ICANS (15%)	(52)
	Improve potency and phenotypic attributes	BMS-986354 (NEX-T process)	60 RRMM	DOR 10.8 Months	ORR (95%)	CRS (82%), ICANS (9%)	(53)

Na, Not available.

GPRC5D is a transmembrane protein with limited expression in normal tissues but selectively highly expressed in MM cells. In 2020, GPRC5D was investigated as a novel target for CAR T-cell therapy in MM (35). The study highlights the toxicity and efficacy of GPRC5D-targeted CAR T-cells in eliminating MM cells in 17 patients with MM. Importantly, targeting GPRC5D did not result in notable off-tumor toxicity, underscoring the specificity and safety of this approach. Dysgeusia is a known side effect of GPRC5D bispecific T-cell engager antibodies. With GPRC5D-targeted CAR T-cells, dysgeusia was seen in only 2 out of 17 patients. CRS and nail changes were seen in 88% and 65% of patients. Overall responses were notable in about 70% of patients, even those who had received prior BCMA-based therapy. This study marks a promising step forward in developing next-generation CAR T-cell therapies for MM (35).

SLAMF7, also known as CS1, is highly expressed on myeloma cells and natural killer (NK) cells. However, SLAMF7 is also expressed on activated T-cells, which raises concerns about CAR-T cell fratricide (54, 55).

Similarly, fratricide is a concern in CD38 CAR T-cells (56). CD38 is another well-established target in MM, with drugs like daratumumab and isatuximab (CD38 monoclonal antibodies). However, CD38 is expressed in normal T/NK cells. CD38-CAR T- cells have shown preclinical success and are being evaluated in early-phase clinical trials (57). CD38 CAR T-cells remains in the early phase of development.

CD138 is highly expressed on plasma cells and is another potential target for CAR T-cell therapy. One major obstacle is CD138 expression on other cell types, including subsets of epithelial and endothelial cells (58). CD138-CAR T-cells have shown promise in preclinical studies and are currently under investigation in clinical trials (NCT03672318).

Dual-targeting CAR T-cells that simultaneously target BCMA and another antigen (e.g., CD19, GPRC5D, CD38, or SLAMF7) are under development. These bispecific CAR T-cells aim to prevent antigen escape and increase durable responses by targeting multiple pathways simultaneously. One innovative strategy to enhance the efficacy of CAR T-cell therapy is the combination of CD19 and BCMA, which has shown significant promise among the various dual-targeting strategies.

CART-ddBCMA is another unique BCMA CAR-T product with a D-binding Domain, comprising 73 amino acids, and offering a highly stable bond with reduced immunogenicity. The CART-ddBCMA showed an ORR of 100% in a phase 1 trial (59). Responses were deep where 22 of the 37 patients achieved sCR/CR, 7 VGPR, and 2 PR. Of 22 patients who were evaluable for MRD, 19 were MRD negative at 10^-5^ or lower (59).

CD19 is a protein commonly found on B cells, and has been successfully targeted in B-cell malignancies such as acute lymphoblastic leukemia (ALL) and non-Hodgkin lymphoma (NHL). Although CD19 is not typically found on myeloma cells, it is present in a subset of early B-lineage precursors and plasmablasts that can eventually develop into myeloma cells (9, 60). This dual-targeting approach addresses the limitations of single-target CAR T-cell therapies by targeting both malignant plasma cells and their progenitors. Both preclinical studies and early-phase clinical trials have investigated CAR T-cells’ effectiveness in targeting CD19 and BCMA. Early-phase clinical trials have shown promising results. For example, a study by Zhao et al. reported the outcomes of a phase 1/2 trial that treated 21 patients with dual-targeting CD19 and BCMA CAR T-cells. The combination achieved an overall response rate of 95% in infused patients, with 43% of patients experiencing complete remissions (41).

The future of CAR T-cell therapy for MM is promising, with ongoing research focused on identifying and validating new targets beyond BCMA. Antigens such as GPRC5D, SLAMF7, CD38, and CD138 represent exciting avenues for exploration, offering hope for more effective and durable treatments. As these novel CAR T-cell therapies advance through clinical trials, they hold the potential to transform the landscape of MM treatment, providing new options for patients who have exhausted current therapies.

## 4 Potential mechanisms of resistance to approved CAR T-cells

The mechanisms of resistance to CAR T-cells have not been fully elucidated. In this section, we describe some of the proposed mechanisms. Because these patterns of resistance are multifactorial, we divided them into the following:

### 4.1 Tumor intrinsic mechanisms of resistance

#### 4.1.1 Bi allelic loss of *BCMA*

It is a rare event and accounts for about 6% of the cases at the time of relapse (61). Bi allelic loss of BCMA was first described by Samur et al. in a patient who received ide-cel at a dose of 150x10^6^ CAR+ T cells following 4 prior lines of therapy (including a proteasome inhibitor, an immunomodulatory agent, and an anti-CD38 monoclonal antibody) (62). The patient achieved a partial response; nonetheless, the disease relapsed at 9 months post CAR T-cells, which necessitated a second infusion of ide-cel at a higher dose (450x10^6^). Unfortunately, the patient derived no clinical benefit from the second dose. The lack of response evoked further investigation to help uncover the underlying mechanisms of resistance. Transcriptomic analysis revealed deletion of 16p in the majority of the multiple myeloma cells; it is worth noting that the BCMA gene (*TNFRSF17*) is located on 16p13.13. This finding was further substantiated by whole exome sequencing of purified CD138+ cells which also identified the presence of a loss of function mutation (p.Q38*) in BCMA in 70%. The authors attributed this lack of response to a lack of BCMA expression secondary to the biallelic loss of BCMA (monoallelic loss of 16 p and second copy loss-of-function mutation), which provides the molecular basis for the lack of BCMA expression in MM cells at the time of relapse. Da Via MC also reported a case of homozygous (biallelic) BCMA gene loss or deletion in a 71-year-old male who received ide-cel at a dose of 450x10^6^. At the time of disease progression, BCMA expression was undetectable compared to baseline (P<6.2×10−94) (63).

#### 4.1.2 Gamma secretase production

BCMA can be cleaved from the surface of myeloma cells by the protease, gamma-secretase, thereby increasing the concentration of soluble/circulating BCMA in plasma (64). The reduced density of surface BCMA may in fact compromise the activity of BCMA targeting therapies such as ide-cel and cilta-cel. Indeed, the study by Chen et al. demonstrated that circulating BCMA levels exceeding 156 ng/mL in samples obtained from 379 patients with RRMM were associated with reduced binding of anti-BCMA antibodies to myeloma cells (65). Hence, inhibition of γ-secretase activity could increase the efficacy of anti-BCMA CAR T-cell therapy via upregulation of BCMA density on plasma cells (66). The combination of a gamma-secretase (crenigacestat) with BCMA targeting CAR T-cells was evaluated in the phase I study of Cowan et al. (46) Of the 18 patients included in the study, 7 had prior exposure to BCMA-targeted therapy. Administration of 3 doses of crenigacestat of 25 mg every other day prior to lymphodepleting chemotherapy resulted in an increase in the BCMA binding sites in all patients, including those with prior BCMA-targeted therapies. The ORR in this subgroup of patients (prior to BCMA-targeted therapy) was 71%, with 43% achieving at least a very good partial response. However, the PFS was shorter (2.6 months) compared to 28.8 months among those with no prior BCMA-targeted targeted therapies. Given the small number of patients, larger studies are needed to validate the efficacy of this approach.

### 4.2 T-cell exhaustion mechanisms

The expansion kinetics of CAR T-cells are crucial for effectively eliminating tumor cells. The success of CAR T-cell therapy is closely linked to the fitness and memory-like characteristics of the cells (67). Less-differentiated T-cells exhibit robust proliferative potential and resistance to exhaustion, with decreased expression of inhibitory receptors such as checkpoint inhibitors, leading to enhanced CAR T-cell expansion. Achieving optimal CAR T-cell expansion by day +7 was an independent and dynamic indicator of treatment response (68). In the early phase 1 clinical trial of cilta-cel, the CAR T-cells were undetectable in the peripheral blood of most patients at four months; the CAR T-cells persisted up to 10 months in merely 16% of the patients (69). CAR T-cell persistence at 3 and 12 months was noted in 86% and 20% of the patients treated with ide-cel respectively (70).

Myeloma cells can evade elimination by CAR T-cells, even if they still have the BCMA target antigen. This is due to CART-cell exhaustion or alteration of the internal apoptotic machinery in plasma cells (71). CAR T-cell exhaustion can happen because plasma cells interact with inhibitory ligands on T-cells (like TIGIT, TIM3, and/or LAG3), or over express PDL1 or lack the expression of costimulatory ligands like CD58 on plasma cells (67). Additionally, using CAR T-cell therapies in earlier lines of therapy was associated with impressive overall response rates and progression-free survival (KarMMa-3 and CARTITUDE4).

Several patient-related factors could impact CAR T-cell efficacy. For instance, using alkylating agents prior to lymphocyte collection may hinder CAR T-cell fitness and decrease the CD4+/CD8+ ratio, while using selinexor might improve lymphocyte fitness (72–74). Even after lymphocyte collection, bridging chemotherapy might affect the absolute lymphocyte count (ALC) before lymphodepletion chemotherapy and modulate CAR T-cell efficacy. The response after CAR T-cell therapy was significantly higher in patients with a high pre-lymphodepletion ALC, which was defined as 0.75 ≥ 10^9/L (76% versus 41%; P = .002). Patients with a low pre-lymphodepletion ALC had a significantly inferior OS (15.4 months) and PFS (8.4 months) compared with those with a high pre-lymphodepletion ALC (OS: not reached, p<0.001; PFS: 27.3 months, <0.001). Notably, higher pre-lymphodepletion ALC was not correlated with higher CRS rates (73). Alternatively, one study demonstrated that a higher baseline ALC was associated with higher CRS/ICANS rates; however, this did not translate into improved survival rates (75). Regardless of the baseline ALC, Saldarriaga et al. showed that the maximum ALC within 15 days after the CAR-T infusion at a dose of 1.0 x 10^3/uL was an independent predictor of PFS (76). These conflicting findings require validation in future studies to better understand the contribution of ALC to patient outcomes and complications with CAR T-cells.

Additionally, underlying comorbidities may also play a role in determining CAR T-cell eligibility. A retrospective analysis indicated that the presence of renal impairment was associated with prolonged cytopenias (77). With advancing age, there is an accumulation of antigen-experienced and dysfunctional T-cell subsets, such as TEMRA, TEX, and TTD cells, as well as selective retention of antigen-inexperienced T cells with memory-like features and NK cell-like markers (67, 78). Data from the CIBMTR registry suggested that there were no significant differences in ORR, median OS, and treatment-related mortality between patients aged 70 or older and younger patients who received ide-cel (79). Regarding toxicity, the older population exhibited higher rates of low grade (1 and 2) neurotoxicity; rates of severe neurotoxicity (grade 3 and above) was comparable between the two age groups. The study also looked at frail patients with score of ≥2 using the simplified frailty index. Frail patients showed similar PFS and OS, but had higher rates of prolonged cytopenia, clinically significant infections, and neurotoxicity of any grade without an increase in high-grade adverse events (79).

Patient-related factors could be modifiable and significantly impact CAR T-cell fitness. According to a prospective pilot study, a six-month physical activity intervention led to notable reductions in levels of T-cell exhaustion markers such as PD-1, TIGIT, TIM3, and/or LAG3 at the end of the intervention compared to the baseline (80).

### 4.3 FDA warnings about CAR T-cells

The FDA utilized the Adverse Event Reporting System (FAERS) to uncover a significant risk of T-cell malignancies linked to all currently approved BCMA-directed and CD19-directed genetically modified autologous CAR T- cell immunotherapies. As a result, in January 2024, the FDA implemented safety labeling revisions across this therapeutic class.

The analysis involved 12,394 adverse event records related to CAR T-cell therapy in the FAERS database. Among these entries, 536 (about 4.3%) documented secondary primary malignancies along with other adverse reactions. Axicabtagene ciloleucel was associated with 51.7% of these cases, while tisagenlecleucel was linked to 33%. However, -ide-cel and cilta-celcomprised 4% and 3% of the reported cases, respectively (81).

The most frequently observed secondary cancers after CAR T-cell therapy were leukemias, comprising 2.7% of all reported incidents. Skin cancers emerged as the second most common, accounting for 0.4% of the overall adverse event reports. Additionally, seventeen instances of T-cell non-Hodgkin lymphomas were recorded, predominantly featuring anaplastic large T-cell lymphomas. Furthermore, two cases of large granular T-cell leukemia were identified, bringing the total T-cell malignancy reports within the FAERS database to 19.

## 5 Discussion

The treatment landscape of RRMM is ever evolving with the identification of new targets and the development of novel treatment modalities. The addition of ide-cel and cilta-cel CAR T-cells to the therapeutic armamentarium of multiple myeloma translated into dramatically improving patient outcomes. The LocoMMotion study demonstrated that standard therapies have minimal activity in triple-class exposed patients (i.e. received an immunomodulatory agent, a proteasome inhibitor, and an anti-CD38 monoclonal antibody) where response rates were approximately 30% (95% CI: 24.2–36.0) (82). Additionally, median PFS and OS were 4.6 (95% CI: 3.9–5.6) and 12.4 months (95% CI: 10.3–NE), respectively. CAR T-cells, as well as other T-cell redirection therapies, were developed to address this urgent or unmet demand for more potent and effective therapies for heavily pretreated multiple myeloma patients. Although CAR T-cell therapy has significantly increased response rates by 2 to 3-fold, the disease will inevitably relapse as treatment-resistant clones start to emerge. As discussed earlier, our deeper understanding of the molecular mechanisms that confer resistance to CAR T-cells has set the framework for some of the clinical trials listed herein. If these investigational CAR T-cells are granted approval, this will pose great challenges to the treating physician as treatment options for RRMM continue to grow. Hence, further research is still needed to not only better sequence these agents but also identify the optimal strategy which can alter the natural history of the disease and thereby lead to a cure.

## Author contributions

SA: Writing – original draft, Writing – review & editing. IH: Writing – original draft, Writing – review & editing. RF: Writing – original draft, Writing – review & editing. SM: Writing – original draft, Writing – review & editing, Conceptualization, Data curation, Formal Analysis, Funding acquisition, Investigation, Methodology, Project administration, Resources, Software, Supervision, Validation, Visualization.
